# Supplementary material for: Implications of variable synaptic weights for rate and temporal coding of cerebellar outputs
Source: bioRxiv. 2023 May 25:2023.05.25.542308. Preprint. [Version 1] doi: 10.1101/2023.05.25.542308 (PMC10245953; doi:10.1101/2023.05.25.542308)
Supplement: Supplement 1 [file NIHPP2023.05.25.542308v1-supplement-1.pdf]

bioRxiv preprint doi: https://doi.org/10.1101/2023.05.25.542308; this version posted May 25, 2023. The copyright holder for this preprint (which was not certified by peer review) is the author/funder, who has granted bioRxiv a license to display the preprint in perpetuity. It is made available under aCC-BY 4.0 International license.

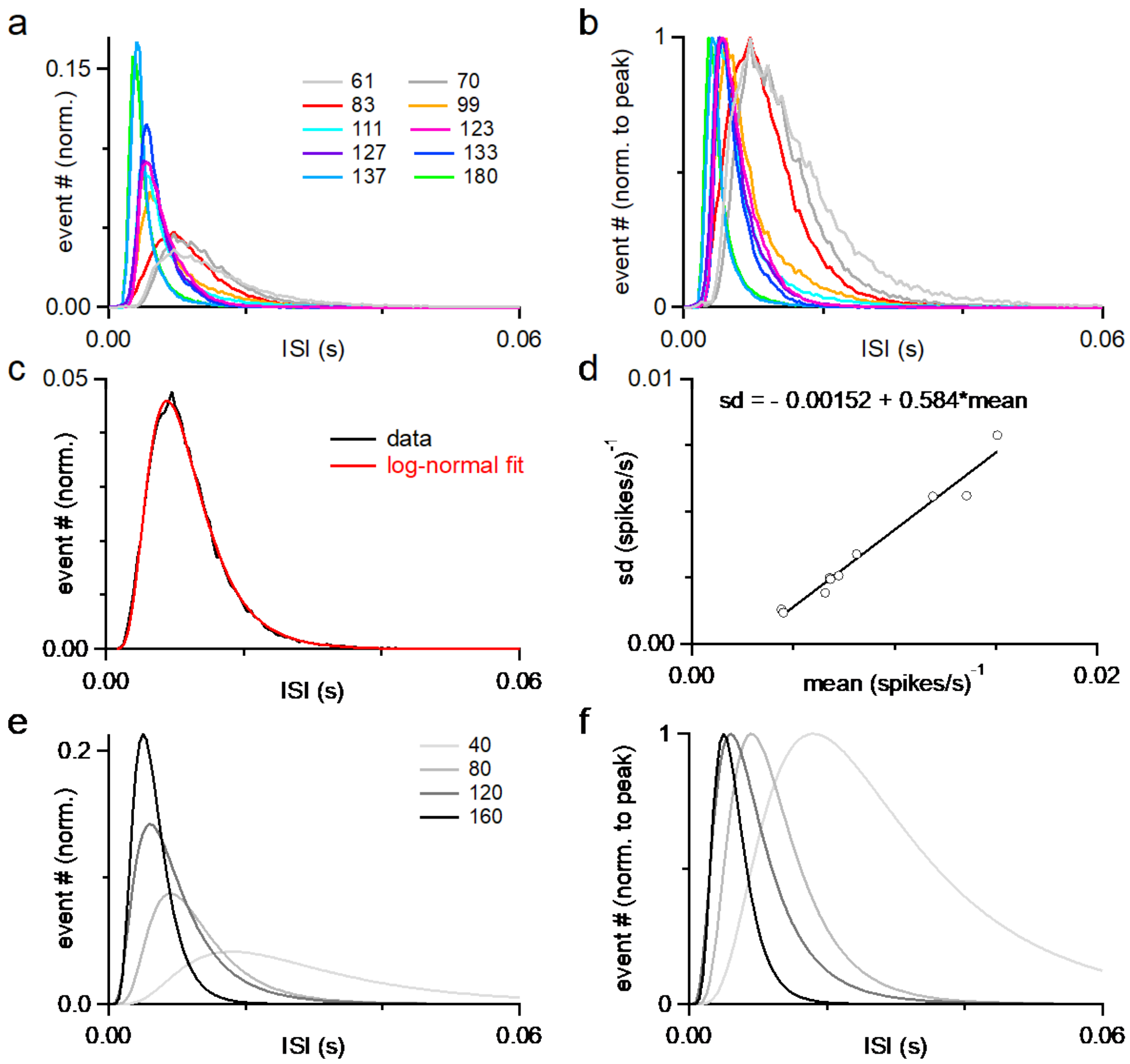

Figure 2—figure supplement 1. ISI histograms of PCs firing used in this study.

- **a.** Normalized ISI histograms of 10 PCs recorded *in vivo*, with their firing rates indicated in the legend.
- **b.** As in **a** but normalized to the peak.
- **c.** Example of a lognormal distribution fitting (red) to the ISI histogram of one PC recoded *in vivo* (black, 83 Hz). Similar fittings were performed for the other 9 PCs.
- **d.** The standard deviation (sd) as a function of the mean of the lognormal distribution fits to the 10 PCs was fitted as a linear function. Fits were performed in IGOR Pro to the function $exp\{-[ln(x/x_0)/width]^2\}$. The values of μ and σ for standard lognormal distribution are computed from x_0 and width using equations: μ = $\ln(x_0) + width^2/2$, σ = $width/\sqrt{2}$. The values of mean and sd of the lognormal distribution fits were computed from μ and σ using equations: mean = $exp(\mu + \sigma^2/2)$ and sd = $\sqrt{exp(2\mu + \sigma^2)\ [\exp(\sigma^2) - 1]}$. This linear function was used to determine the parameters for a PC firing ISI lognormal distribution with a desired firing rate.
- **e.** Four lognormal distributions representing artificial PC firing ISI distributions with different firing rates generated with the mean and sd from the linear function in **d**.
- **f.** As in **e** but normalized to the peak.
